# Air gap technique is recommended in axiolateral hip radiographs

**DOI:** 10.1002/acm2.13021

**Published:** 2020-09-21

**Authors:** Susanne Kivistö, Antti Kotiaho, Anja Henner, Terhi Nevala, Jaakko Niinimäki, Miika T. Nieminen, Matti Hanni

**Affiliations:** ^1^ Department of Diagnostic Radiology Oulu University Hospital Oulu Finland; ^2^ Research Unit of Medical Imaging Physics and Technology University of Oulu Oulu Finland; ^3^ Medical Research Center University of Oulu and Oulu University Hospital Oulu Finland; ^4^ Oulu University of Applied Sciences Oulu Finland

**Keywords:** air gap, CDRAD phantom, grid, hip axiolateral projection, radiography

## Abstract

**Purpose:**

To investigate the replacement of conventional grid by air gap in axiolateral hip radiographs. The optimal air gap distance was studied with respect to radiation dose and image quality using phantom images, as well as 26 patient axiolateral hip radiographs.

**Methods:**

The CDRAD phantom, along with polymethylmethacrylate slabs with thicknesses of 10.0, 14.6, and 20.0 cm was employed. The inverse image quality index and dose area product (DAP), as well as their combination, so called figure‐of‐merit (FOM) parameter, were evaluated for these images, with air gaps from 20 to 50 cm in increments of 10 cm. Images were compared to those acquired using a conventional grid utilized in hip radiography. Radiation dose was measured and kept constant at the surface of the detector by using a reference dosimeter. Verbal consent was asked from 26 patients to participate to the study. Air gap distances from 20 to 50 cm and tube current‐time products from 8 to 50 mAs were employed. Exposure index, DAP, as well as patient height and weight were recorded. Two radiologists evaluated the image quality of 26 hip axiolateral projection images on a 3‐point nondiagnostic — good/sufficiently good — too good scale. Source‐to‐image distance of 200 cm and peak tube voltage of 90 kVp were used in both studies.

**Results and conclusion:**

Based on the phantom study, it is possible to reduce radiation dose by replacing conventional grid with air gap without compromising image quality. The optimal air gap distance appears to be 30 cm, based on the FOM analysis. Patient study corroborates this observation, as sufficiently good image quality was found in 24 of 26 patient radiographs, with 7 of 26 images obtained with 30 cm air gap. Thus, air gap method, with an air gap distance of 30 cm, is recommended in axiolateral hip radiography.

## 1 INTRODUCTION

Air gap technique is a well‐known method to reduce the amount of scattered x‐ray radiation reaching the detector, thus reducing noise and improving image contrast.^1^ It is rather commonly utilized instead of a conventional grid in plain radiography.^2^, ^3^, ^4^, ^5^, ^6^ Air gap is an additional distance between a patient and an image detector.^7^ The gap decreases the likelihood for scattered x‐ray radiation to reach the detector, as radiation is partially absorbed and scattered in the air.^8^, ^9^ Air gap technique offers advantages over conventional grids, as the latter may, for example, lead to image artifacts, typically related to the misalignment of the grid. In the air gap technique, the object to image distance (OID) is increased compared to the imaging with a conventional grid, which results in magnified image.^8^, ^9^ To reduce magnification, source to image distance (SID) can be increased, albeit in some cases imaging geometry may pose limitations for this increase. In recent years, so called virtual grid techniques have also been proposed.^10^, ^11^ These techniques model scattered radiation mathematically, and at least partially remove its effects from the image to enhance contrast.

Early studies on air gap methods concentrated on chest radiographs.^12^, ^13^ These studies indicate that images acquired with air gap technique can provide contrast similar to images acquired using conventional grids, in particular for the posterior–anterior projection^12^ with no increase in the patient exposure. Later, Chan and Fung^14^ reported 10 cm to be the optimal air gap distance at pelvic anterior–posterior (AP) examination. They reported that the effective dose was reduced by a factor of roughly two in both computed radiography (CR) and digital radiography (DR) examinations by replacing antiscatter grid with an air gap of 10 cm, while image quality was observed to be diagnostic. On the contrary, Moey *et al*. reported that pelvic radiographs obtained with air gap distances <20 cm were not fully acceptable.^15^ Trimble^16^ assessed image quality of both lateral thoracic spine and chest radiographs using conventional grid, as well as air gap techniques and concluded that image quality was higher with the latter. Bell *et al*.^5^ questioned the use of conventional grid on slim to average‐weighted patients in cervical spine lateral projection, as the air gap technique sufficiently reduces scattered radiation and provides diagnostic images. On the other hand, Keating and Grange^4^ recommended incorporation of grid for adult AP cervical spine radiography, based on the results on the radiography of a lamb neck. Partridge *et al*.^17^ suggested using an air gap of 15 cm instead of a grid as the default method for coronary angiography and intervention.

In horizontal beam lateral or axiolateral hip projection, the air gap technique has been shown to reduce radiation dose, and demonstrates diagnostic image quality, as compared to the use of conventional grid.^18^, ^19^ At our institution, examinations of the hip comprise roughly 5% of all conventional radiographic studies. Most examinations involve the axiolateral projection with horizontal x‐ray beam, a common protocol for patients with hip trauma. This projection has also been employed to calculate cup anteversion after hip arthroplasty surgery.^20^, ^21^, ^22^

The aim of the study was to investigate whether the conventional grid can be replaced by an air gap technique in the hip axiolateral radiographic projection. The optimal air gap distance was sought by acquiring images with a contrast‐detail phantom with different air gap distances. Both image quality and dose area product (DAP), as well as their combination, so called figure‐of‐merit (FOM) parameter were utilized in the analysis. A small sample of patient hip axiolateral radiographs were analyzed in terms of air gap distances to verify the results found in the phantom study.

## 2 MATERIALS AND METHODS

This study was performed in two phases; first phase comprised of phantom measurements, and the second phase comprised of a patient study (institutional review board approved the study, 183/2019). All images were obtained with Fuji FDR Acselerate system (Fujifilm Corporation, Tokyo, Japan) with wireless flat panel detectors using cesium iodide (CsI) scintillator (D‐EVO plus C35i). The imaging system is under a periodic quality control program. Total filtration of 2.8 mmAl + 0.1 mmCu and focus size of 1.0 mm were utilized throughout the study. DAP values were measured with an integrated KERMAX‐plus DAP meter (IBA dosimetry GmbH, Schwarzenbruck, Germany). DAP meter has an estimated measurement uncertainty of 4.5% with the peak voltage of 90 kVp with a total filtration of 2.8 mmAl + 0.2 mmCu, using a reference dosimeter (Unfors RaySafe Xi base unit with R/F detector and DXR+, Unfors Raysafe AB, Billdal, Sweden). For this value of uncertainty, however, different amount of copper filtration is utilized than in the actual measurements.

**Fig. 1:**
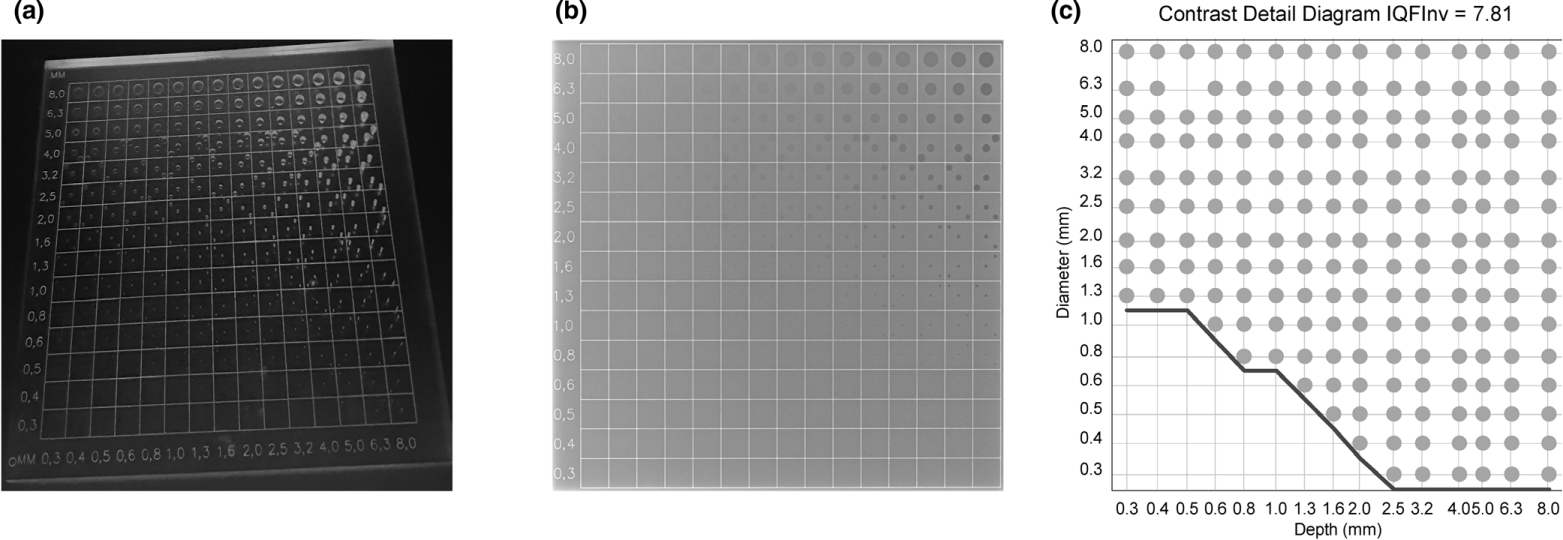
(a) An image of the CDRAD phantom, (b) a representative x‐ray image of the CDRAD phantom, (c) a contrast‐detail curve, from which the inverse image quality index (IQF_inv_) is calculated, obtained as a result of an analysis by software (CDRAD Analyser ver 2.1.15) for (b).

In the phantom study, CDRAD 2.0 phantom (Artinis, Medical Systems B.V., Zetten, The Netherlands) was utilized. CDRAD phantom consists of polymethylmethacrylate (PMMA) square plate (26.5 cm × 26.5 cm) with a thickness 10.0 mm. It has cylindrical holes in 15 rows and 15 columns with both depth and diameter ranging from 0.3 to 8 mm, as shown in Fig. 1(a). Furthermore, a representative x‐ray image and a contrast‐detail curve of the phantom, based on a software analysis (CDRAD Analyser ver 2.1.15^23^) are presented in Figs. 1(b) and 1(c), respectively. A description on the detection methods of the differently sized holes in the phantom, as well as on the resulting contrast‐detail curve is presented in the literature.^23^

**Fig. 2:**
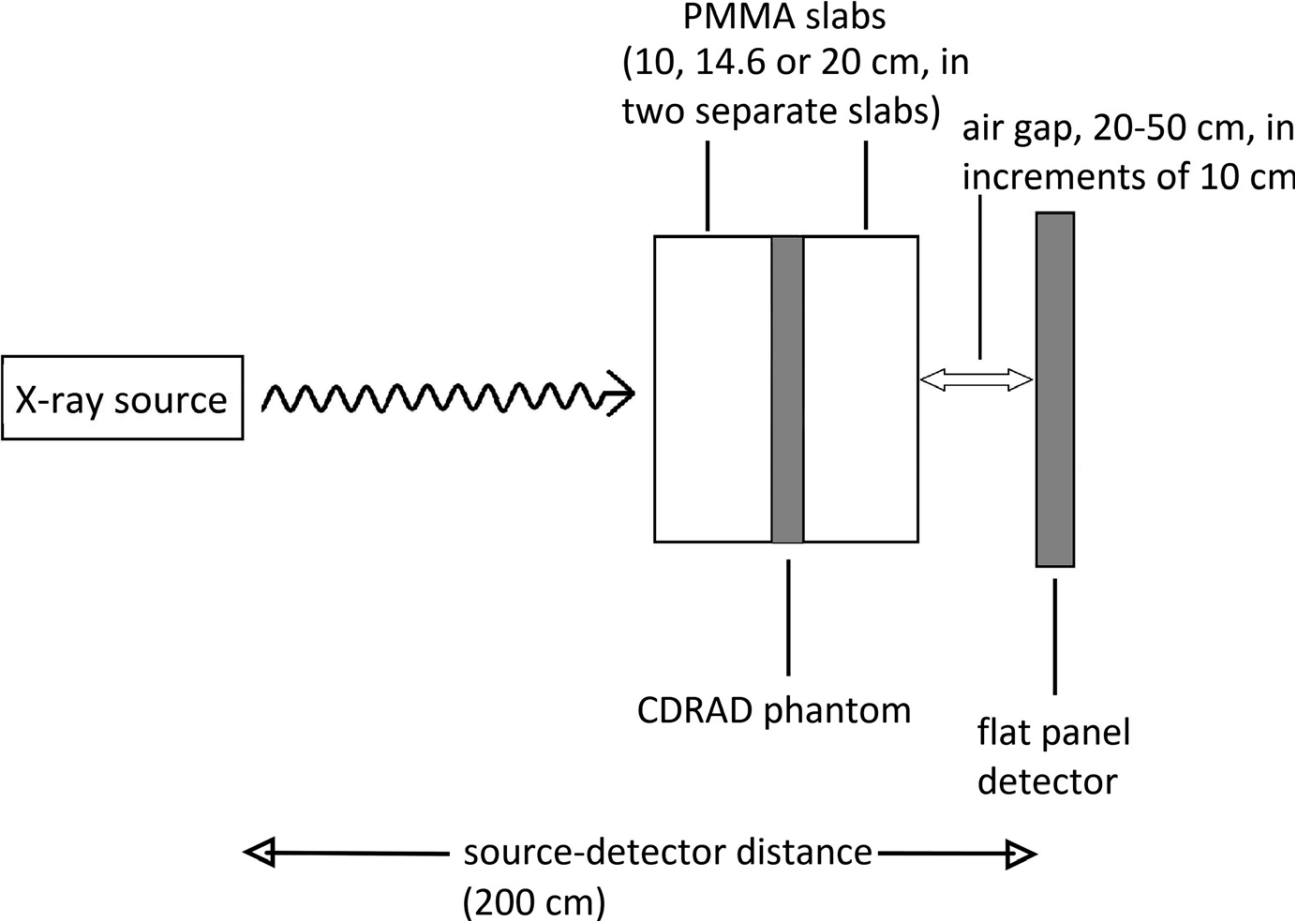
Schematic view of CDRAD phantom measurement setup with different air gap distances and PMMA slab thicknesses.

In phantom measurements, dose at the surface of the detector was set to roughly 20 µGy by manually adjusting tube current‐time product for each imaging setup. A reference dosimeter (Unfors RaySafe Xi base unit with R/F detector, Unfors Raysafe AB, Billdal, Sweden) was employed. All measurements were performed with 90 kVp and SID of 200 cm. PMMA slabs with total thicknesses of 10.0, 14.6, and 20.0 cm were utilized to simulate x‐ray radiation attenuation and scatter in patients’ thighs. The CDRAD phantom was placed in the middle of two individual PMMA slabs, which together yield the total PMMA thickness (see Fig. 2). Images obtained with a parallel antiscatter grid with 215 lines per inch and a grid ratio of 8:1 (Reina Imaging, Crystal Lake, USA) were compared to images acquired with air gaps between 20 to 50 cm in increments of 10 cm.

For each measurement setting, we exposed CDRAD phantom three times. Inverse image quality parameter IFQ_inv_ was determined^23^ as an average of three images. IQF_inv_ is a measure of both image contrast and resolution, and is defined as

$${\text{IQF}}_{\text{inv}}=\frac{100}{\sum_{i=1}^{15}C_{i}D_{i}},$$

where *C_i_* and *D_i_* denote contrast and threshold diameter in column *i*, respectively. A higher IQF_inv_ indicates higher image quality as more and smaller objects are detected from the CDRAD phantom images. The DAP values were calculated as averages from the three exposures as well. IQF_inv_ and DAP values were determined for all PMMA slab thicknesses and air gap distances. In order to probe the image quality with respect to dose, we utilize so called FOM parameter.^24^, ^25^ We replaced the radiation dose quantities used in these publications by DAP, as in

$$\text{FOM}=\frac{{\text{IQF}}_{\text{inv}}^{2}}{\text{DAP}}.$$

We took the effect of dose on image quality into account and calculated FOM parameter for all cases. Furthermore, errors are estimated as standard deviations for DAP and IQF_inv_ values. For FOM, partial derivatives method for error estimation is used.

In the patient study, verbal consent was asked from all 26 patients to participate in the study. SID of 200 cm and peak tube voltage of 90 kVp were set for every patient. Standard patient positioning for axiolateral hip radiographs was carried out. Air gap distances of 20, 30, 40, and 50 cm, as well as tube current‐time products ranging from 8 to 50 mAs were utilized, depending on the radiographers’ subjective evaluation. Furthermore, x‐ray field size changed due to the variation in patient size. A portable flat panel detector without automatic exposure control (AEC) was employed throughout the patient study. Fujifilm uses S value to indicate the radiation exposure to the detector. S value, which is inversely proportional to the radiation exposure on the detector,^26^ was collected for all patient images. DAP values were also collected. Patient height and weight were recorded as well. Two experienced radiologists independently evaluated the quality of the 26 hip axiolateral projection images on a 3‐point nondiagnostic — good/sufficiently good — too good scale. The following criteria were used by the radiologists to score the images. For nondiagnostic images, scatter noise is pronounced and bony interfaces are blurred. Furthermore, joint line of the hip joint, or cortical margins of the femoral neck are not visible. For good/sufficiently good images, scatter noise is visible but bony interfaces are clear. In addition, joint line of the hip joint and cortical margins of the femoral neck are adequately visible. For images evaluated to be too good, the scatter noise of the image is minimal and bony interfaces and trabecular markings are sharp, as well as all the pelvic bone structures are depicted pronouncedly sharp. To test the interobserver reliability, we employed Cohen’s kappa test, as described in Ref. [27].

## 3 RESULTS

**Fig. 3:**
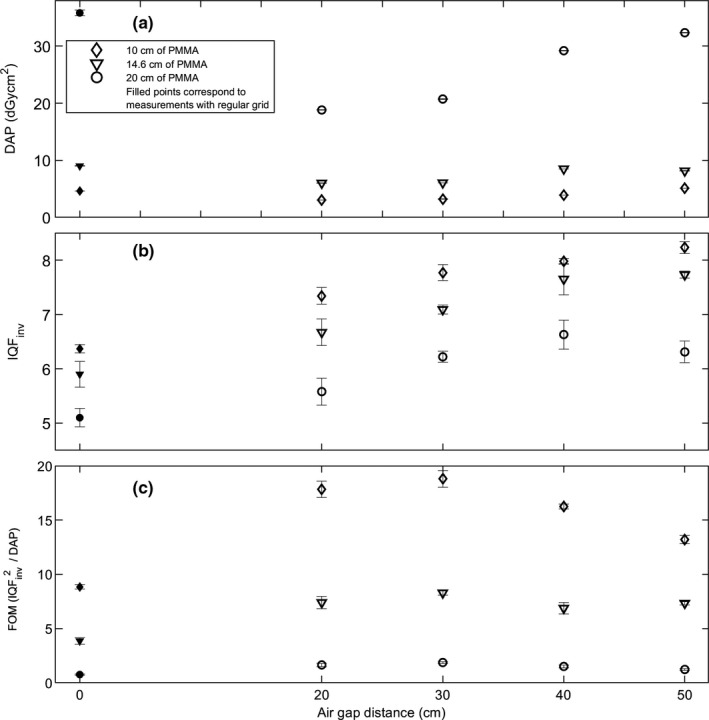
CDRAD phantom measurements containing either conventional grid (filled markers) or air gaps of 20 to 50 cm (unfilled markers), with three different PMMA slab thicknesses (10, 14.6 and 20 cm). Graphs contain (a) dose area products (DAPs), (b) inverse image quality index (IQF_inv)_, and (c) figure‐of‐merit (FOM) parameter in different phantom measurement setups. The error bars are standard deviations from individual measurements (DAP) and analyses (IQF_inv_), as well as error estimates from the use of partial derivatives method for FOM.

In CDRAD phantom measurements (see Fig. 3), using a total of 20.0 cm of PMMA, the DAP was 35.8 dGycm^2^ with the conventional grid. With an air gap of 20 cm, the DAP was reduced by 47% from 35.8 to 18.81 dGycm^2^. When further increasing the air gap, DAP increased, but remained below the value obtained using the conventional grid. For the air gap of 50 cm, the DAP is reduced 10% to value of 32.3 dGycm^2^ as compared to the case of using conventional grid. With PMMA slabs of 10.0 and 14.6 cm, DAP reductions of roughly 6‐40% were observed air gaps of 20–30 cm. With only single exception with a case of 10.0 cm PMMA slab with an air gap of 50 cm, all DAP values were lower with the air gap technique than with the conventional grid [see Fig. 3(a)].

CDRAD Analyser contrast‐detail curve analysis yielded the image quality parameter IQF_inv_. With the conventional grid technique using 10.0 cm PMMA slab thickness, IQF_inv_ was roughly 6.4 [see Fig. 3(b)]. By using air gaps from 20 to 50 cm, IQF_inv_ increased from 7.3 to 8.2. For the 14.6‐cm‐thick PMMA slab, the respective IQF_inv_ values ranged from 5.9 to 7.7. Thus, image quality parameter IQF_inv_ yields no maxima at air gap distances from 20 to 50 cm for PMMA slabs of 10.0 or 14.6 cm. This indicates that even larger air gap distances might be utilized for higher image quality, which would, albeit not in all cases, result in higher DAP values as well [Fig. 3(a)]. On the contrary, in the case of 20.0 cm PMMA slab, IQF_inv_ yielded a maximum value of 6.6 at an air gap of 40 cm. Furthermore, IQF_inv_ decreased from 6.6 to 6.3 when air gap distance increased from 40 to 50 cm. This is might be due to the amount of primary radiation reaching the detector is starting to decrease at the latter distance. Both DAP and IQF_inv_ generally increased as a function of air gap distance for all PMMA slab thicknesses, with only few exceptions (*vide infra*). FOM values increase by up to 150% between the case of conventional grid and air gap distance of 30 cm, and then decrease by up to 57% between air gap distances of 30 and 50 cm, at different PMMA slab thicknesses. A maximum FOM at an air gap distance of 30 cm is observed regardless of the PMMA slab thickness [see Fig. 3(c)].

For the radiographic hip axiolateral projections of 26 patients, the average height and weight of the patients were 164 cm (range 150–182 cm) and 70 kg (50–95 kg), and the average body mass index (BMI) was 26.1 kg/m^2^ (35.6–19.8 kg/m^2^) (see Table 1). The average DAP and tube current‐time product for the patient population were 4.06 dGycm^2^ (0.70–11.30 dGycm^2^), and 26 mAs (8–50 mAs), respectively. The average S value was 773 (277–3565). The air gap varied from 20 to 50 cm, the average being 35 cm.

**Table 1:** Patient BMI, height, and weight, parameters related to image quality and radiation dose, as well as diagnostic evaluations of patient hip axiolateral radiographs.

Patient	BMI (kg/m^2^)	Height (cm)	Weight (kg)	DAP (dGycm^2^)^a^	Tube current‐time product (mAs)	S value^b^	Air gap (cm)	Diagnostic evaluation^c^		
								Too good	Good/sufficiently good	Nondiagnostic
1	27.9	155	67	1.56	20	817	25		o,x	
2	20.8	170	60	1.30	20	620	20		o,x	
3	23.7	163	63	1.80	20	409	30	o	x	
4	31.9	168	90	1.40	20	1294	40		o,x	
5	35.6	150	80	2.00	20	3565	30		o	X
6	22.2	150	50	2.20	20	780	40		o,x	
7	25.1	162	66	4.00	25	605	40		o,x	
8	26.0	182	86	3.65	40	895	30		o,x	
9	31.1	170	90	11.30	40	439	30		o,x	
10	25.3	165	69	6.20	40	439	40		o,x	
11	27.0	170	78	3.20	20	1325	40		o,x	
12	27.0	170	78	2.80	32	1180	40		o,x	
13	20.8	155	50	6.39	25	303	50		o,x	
14	28.2	153	66	4.21	25	409	50		o,x	
15	26.1	176	81	4.04	25	459	40		o,x	
16	33.2	160	85	8.28	32	439	50		o	X
17	21.3	165	58	3.46	20	277	20		o,x	
18	25.6	157	63	4.70	20	340	40		o,x	
19	27.5	165	75	6.64	40	481	40		o,x	
20	31.1	172	92	4.46	32	552	30		o,x	
21	19.8	162	52	0.70	8	1521	20		o,x	
22	31.8	170	92	7.67	50	352	40		o,x	
23	22.6	156	55	3.27	20	578	30		o,x	
24	21.1	163	56	5.40	20	391	44		o,x	
25	23.0	168	65	3.60	32	634	30		o,x	
26	23.5	157	58	1.40	16	982	30		o,x	
Averages	26.1	164	70	4.06	26	773	35			

^a^ Dose area product.

^b^ Exposure index, Fujifilm.

^c^ o = evaluation of radiologist 1, x = evaluation of radiologist 2.

The radiologists evaluated the images based on a 3‐point scale: too good — good/sufficiently good — nondiagnostic with the previously mentioned criteria. Radiologist 1 evaluated one image out of 26 to be too good, and all the other images to be either sufficiently good or good. However, acetabulum was not clearly visible in four of these images. Radiologist 2 evaluated all except two images to be good or sufficiently good. The two exceptions were evaluated as nondiagnostic. Both patients had high BMIs of 33.2 and 35.6 (see Table 1), respectively. The calculated interobserver reliability was poor (kappa = −0.026).

## 4 DISCUSSION

The most suitable air gap distance not only involves a reduction in patient dose but also maintains the diagnostic image quality. Thus, a compromise between the two entities is to be sought. Consequently, we employed the FOM parameter to evaluate the image quality with respect to dose. We observed that the image quality, as determined by the IQF_inv_ parameter in the phantom setup, was always higher with any of the air gap distances employed than with the conventional grid. Furthermore, dose was generally reduced when using the air gap method instead of a grid. The largest DAP reductions of up to 47% were observed using air gaps of 20 to 30 cm, as compared to the case of using a conventional grid, for different PMMA slab thicknesses. At larger air gap distances of 40 and 50 cm, DAP is typically reduced by 10 to 20% as compared to the case of conventional grid, with a single exception found at an air gap of 50 cm in the case of 10 cm PMMA. This observation, together with the decreasing FOM trend in Fig. 3(c) for the PMMA slab thickness of 10.0 cm at air gaps from 30 to 50 cm might indicate that air gap distances larger than 30 cm should not be applied for very thin patients. However, we state that the results of the phantom study should not be directly utilized in clinical imaging protocol without proper measurement of patient thicknesses, as the properties of PMMA are not those of human tissue. The optimal image quality, as indicated by largest IQF_inv_ parameter in our phantom setup, was achieved with air gap distances from 40 to 50 cm, depending on the PMMA slab thickness. An increase of roughly 30% in image quality was observed at these air gap distances. A clear maximum for IQF_inv_ parameter was observed at 40 cm using 20.0‐cm‐PMMA thickness, which might be due to the interplay of a suitable air gap distance and scattering in the air and in the PMMA slabs. For other PMMA slab thicknesses, no such IQF_inv_ maximum was observed. On the contrary, FOM yields a maximum at an air gap distance of 30 cm, regardless of the PMMA slab thickness. IQF_inv_ alone does not correspond to optimal image quality at specific air gap distance, as radiation dose modifies the overall image quality, including noise characteristics. The error bars are generally small for all parameters and do not change the overall trends of different parameters as a function of the air gap distance in Fig. 3. Thus, an optimal air gap distance of 30 cm is suggested for hip radiographs based on the FOM analysis in the current phantom setup, also bearing in mind that different FOM parameters hold different sensitivity with respect to radiation dose.^29^

Charnley *et al*. reported an optimal air gap distance of 45 cm while using an anthropomorphic phantom in a similar setup as presented in this work.^2^ However, their study did not include air gaps smaller than the reported 45 cm. Flintham & Snaith^19^ studied horizontal beam lateral view of the hip for patients. Images acquired with the air gap technique were of higher quality than the images obtained with the conventional grid. However, no detailed radiation dose or air gap distance evaluation was performed in their study, as opposed to our current results. Furthermore, a Monte Carlo simulation study also promotes the use of air gap over conventional grids^28^ for lumbar spine radiographs, albeit the air gap distances were only in the range of 0 to 25 cm.

When automatic exposure control (AEC) cannot be used, radiographers (radiologic technologists) manually select different tube current‐time products and air gap distances based on their expertise, patient size, and medical condition. As an example, 4 of 26 hip axiolateral radiographs were acquired with a tube current‐time product of 20 mAs, and an air gap distance of 40 cm, while the patient BMI varied from 22 to 31 kg/m^2^. These radiographs had S values in the range of 340 to 1325, with larger values indicating smaller doses at the detector. In total, the manufacturer’s recommended S values for hip radiographs (100–400) were exceeded in 21 of 26 cases. Furthermore, tube current‐time product value was not always properly selected based on the observed S values and patient BMIs. As an example, two patient images were evaluated as nondiagnostic, as the other one did not show the acetabulum, and in the other only the prosthesis was visible while bones were not. These patients were obese with BMIs of 33.2 and 35.6 kg/m^2^, respectively. Furthermore, the S values for these exposures were 439 and 3565, respectively, along with tube current‐time product values of 32 and 20 mAs. Thus, an improper manual selection of tube current‐time product could have been carried out by the radiographer, at least in the latter case. Despite the differences in S, as well as tube current‐time product values, the radiologists graded most of the images as adequate for diagnostic purposes. Thus, we emphasize that imaging parameters should be carefully selected for each patient and diagnostic image quality is achievable with S values exceeding the recommended range. This is crucial, as the hip axiolateral projection is often imaged using a portable computed or direct radiography detector, and the use of AEC is not always possible.

Use of air gap technique is advisable for many reasons. Misalignment of the grid^30^, ^31^ was obviously absent, as no physical grid was employed. Consequently, the common grid artifacts^32^, ^33^ did not appear in the images. The geometric magnification of the image, resulting from the use of air gap, did not hinder image evaluation for the radiologists, based on our current results.

Despite the positive features of the air gap technique presented above, shortcomings exist in our study. In the phantom setup, more CDRAD phantom images could have been acquired to enhance the statistical reliability of our results. Furthermore, we did not study the temporal response of the detector to reveal the effects of ghosting and lag in the images. In the patient study, some air gap distances yielded mixed results in terms of image quality. As an example, an air gap of 30 cm yielded a too good, as well as a nondiagnostic image. The latter image was for a patient with BMI of 35.6 kg/m^2^ and acquired using a tube current‐time product of 20 mAs. This resulted in underexposed and nondiagnostic image with a DAP of 2.0 dGycm^2^ and an S value of 3565. While one radiologist evaluated that the acetabulum was only weakly visible in this case, the other radiologist stated that it was not visible at all. Patient population was rather small, and no control group formed from the same patients and imaged using a regular grid, was used. Furthermore, patient images were consciously acquired with different air gap distances, based on radiographers’ subjective evaluation, which might modify the diagnostic value of the images. However, 24 of 26 patient images were assessed as diagnostic by the radiologists. As the number of patients was rather small, and there were few differently evaluated images between the readers, the interobserver reliability was very low. No intraobserver differences were recorded, as the radiologists evaluated each image only once. However, based on the overall accuracy of the radiologists’ statements and their relevant work experience, no large changes are expected in the intraobserver reliability. Further work is needed to match different tube current‐time product values to measurements of thighs of different thicknesses in an anthropomorphic phantom to mimic clinical conditions in patient imaging, as well as to ultimately transfer the results of the phantom study to clinical imaging protocol. Also, newly developed virtual grid techniques, in addition to the conventional grid, as well as air gaps, should be evaluated with respect to image quality and dose.

## 5 CONCLUSIONS

Based on the FOM analysis combining the effects of image quality and radiation dose in the current phantom setup, the optimal air gap distance in the axiolateral hip radiographs appears to be 30 cm. With this air gap distance, all patient hip radiographs were evaluated either too good or good/sufficiently good. A single exception occurs for a hip radiograph of an obese patient with insufficient tube current‐time product value. As some of the recorded imaging parameters of the patient study imply, careful, and continuous education on different radiographic techniques in a real clinical environment is essential for diagnostic radiography.

## AUTHORS' CONTRIBUTIONS

SK was involved in conceptualization, data curation, formal analysis, investigation, methodology, and writing — original draft preparation and review/editing; AK was involved in conceptualization, data curation, formal analysis, investigation, methodology, visualization, and writing – original draft preparation and review/editing; AH was involved in conceptualization, methodology, and writing — review and editing; TN was involved in data curation, formal analysis, and writing — review and editing; JN was involved in conceptualization, methodology, resources, data curation, formal analysis, and writing — review and editing; MTN was involved in conceptualization, methodology, project administration, resources, supervision, and writing — review and editing; MH was involved in conceptualization, data curation, formal analysis, investigation, methodology, project administration, supervision, visualization, and writing – original draft preparation and review/editing.

## CONFLICT OF INTEREST

No conflict of interest.
